# An Overview of Recent Clinical Trials for Diabetic Foot Ulcer Therapies

**DOI:** 10.3390/jcm13247655

**Published:** 2024-12-16

**Authors:** Ovya Ganesan, Dennis P. Orgill

**Affiliations:** 1 Department of Plastic Surgery, Brigham and Women’s Hospital, Boston, MA 02115, USA; oganesan@bwh.harvard.edu; 2 Geisel School of Medicine at Dartmouth, Hanover, NH 03775, USA; 3 Harvard Medical School, Boston, MA 02115, USA

**Keywords:** diabetic foot ulcer, therapy, treatment, diabetes, ulcer, diabetic foot

## Abstract

Diabetic foot ulcers (DFUs) are a major complication of diabetes, leading to high mortality, reduced quality of life, neuropathy, ischemia, infection, and amputation risks. The prevalence of these ulcers is only on the rise as more people suffer from type 2 diabetes and obesity. The current wound management involves wound dressings, offloading, debridement, and infection control, but more must be done to keep up with the rising prevalence of DFUs and the strain they put on patients and the healthcare system. To find recent therapeutic advances in DFU treatment, we searched PubMed for novel therapeutics from the past 5 years. We found a diversity of promising interventions, including advanced wound dressings and topicals, physical energy-based therapies, regenerative scaffolds, and growth factor- and cell-based therapies. Recent therapies hold significant promise in healing more DFUs faster and more effectively. Providers should consider employing safe, novel therapeutics when standard dressings are not effective.

## 1. Introduction

Diabetic foot ulcers (DFUs) are a growing concern worldwide, particularly as the prevalence of type 2 diabetes mellitus (T2DM) and obesity rise. DFUs are now one of the most common reasons for hospital admission among individuals with diabetes, representing a significant public health burden [1,2]. The increasing prevalence of obesity, high cholesterol, hypertension, and lack of physical activity are directly correlated with the growing incidence of T2DM. In fact, as of 2021, more than 10% of the United States population had diabetes, and about 30% of adults older than 65 had diabetes [1,2].

DFUs pose severe challenges to patients and the healthcare system due to their associated comorbidities, including neuropathy, ischemia, infection, and high risk of amputation [2]. These complications greatly diminish quality of life, with mortality rates as high as 45% five years after a DFU diagnosis [3].

**Figure 1:**
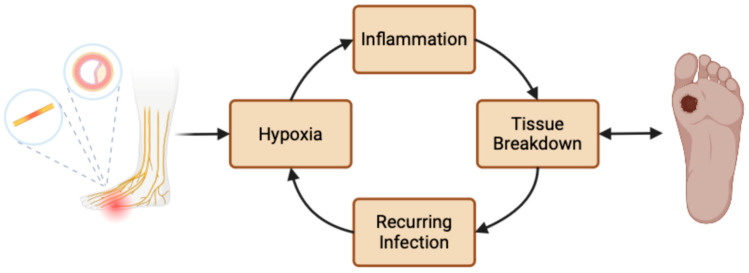
Depiction of several of the factors that allow for the persisting nature of diabetic foot ulcers. Created with BioRender.com.

**Figure 2:**
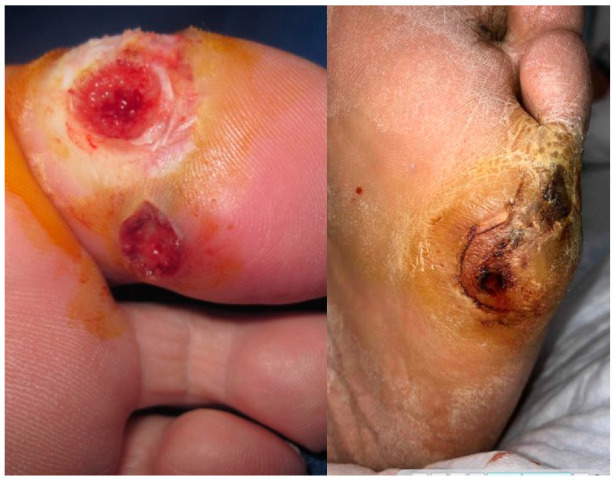
Diabetic foot ulcers.

The pathogenesis of DFUs involves a complex interplay of factors. Atherosclerosis, a common contributing factor of diabetes, reduces blood flow to extremities, depriving tissue of essential oxygen and nutrients needed for healing wounds. Peripheral neuropathy, due to prolonged hyperglycemia that damages blood vessels and nerve tissue, makes patients more susceptible to not noticing small wounds. Subsequently, DFUs are able to flourish into nonhealing, chronic wounds. Their persistence is compounded by increased inflammation, recurring infection, moisture-related skin breakdown, and resulting hypoxia (Figure 1 and Figure 2).

Investigators have aimed to create therapies that target the complex contributing factors and mechanisms that result in DFUs. On top of optimal glycemic control, the current standard treatments for DFUs may include offloading, wound debridement, and the application of dry or moist dressings. Given the rise in DFU prevalence, along with associated comorbidities and strain on the healthcare system, a plethora of therapies have been investigated in recent years. These therapies are widely heterogeneous, however, as there are a myriad of pathways that contribute to DFUs. This heterogeneity, compounded by the lack of comparative studies between therapies, creates a challenge for clinicians in identifying which therapy is best suited for their patients. This review serves to summarize recent advances in therapy and highlight strengths of each therapy.

## 2. Materials and Methods

Due to the diffuse and heterogeneous nature of these publications, a systematic review was not performed. Instead, a general review was conducted; PubMed was searched for articles relating to DFUs and therapies. Key terms included “diabetic foot”, “therapy”, and “standard of care”. Results were filtered to include clinical trials and randomized trials from the past 5 years that were written in English. These study types were included due to their ability to provide high-quality data directly applicable to patient care. Restricting the search to recent studies ensured the avoidance of outdated information while reflecting current practices and emerging trends.

**Figure 3:**
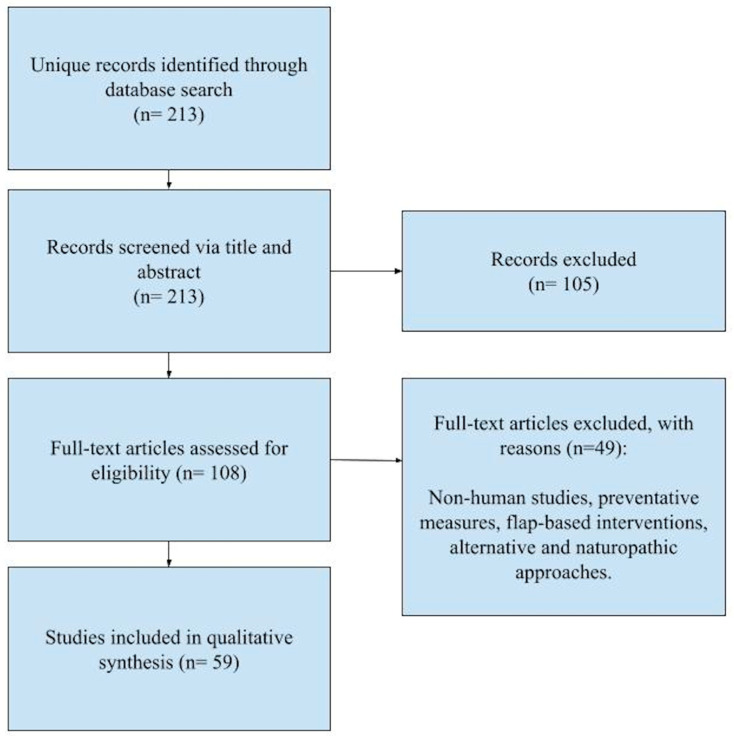
Flow diagram of selected studies.

In total, 213 articles were identified, and after title and abstract screening, 108 articles met inclusion criteria. After full-text screening, 59 articles were included in this review (Figure 3). We excluded studies that looked at lower extremity wounds and neuropathy rather than DFUs specifically (as this review is specific to DFUs); looked at therapies in animals rather than humans (indirect clinical relevance); preventative studies (this review is focused on therapies for existing DFUs); investigated flap- and graft-based interventions; and studied alternative, naturopathic, or traditional approaches (lack of robust clinical evidence supporting validates interventions). These specific exclusion criteria were chosen to reduce heterogeneity of this diffuse topic while limiting to higher-quality, modern evidence.

This approach allowed for an overview of emerging therapeutic strategies. See Supplementary File S1 for a full list of screened articles.

## 3. Results

**Figure 4:**
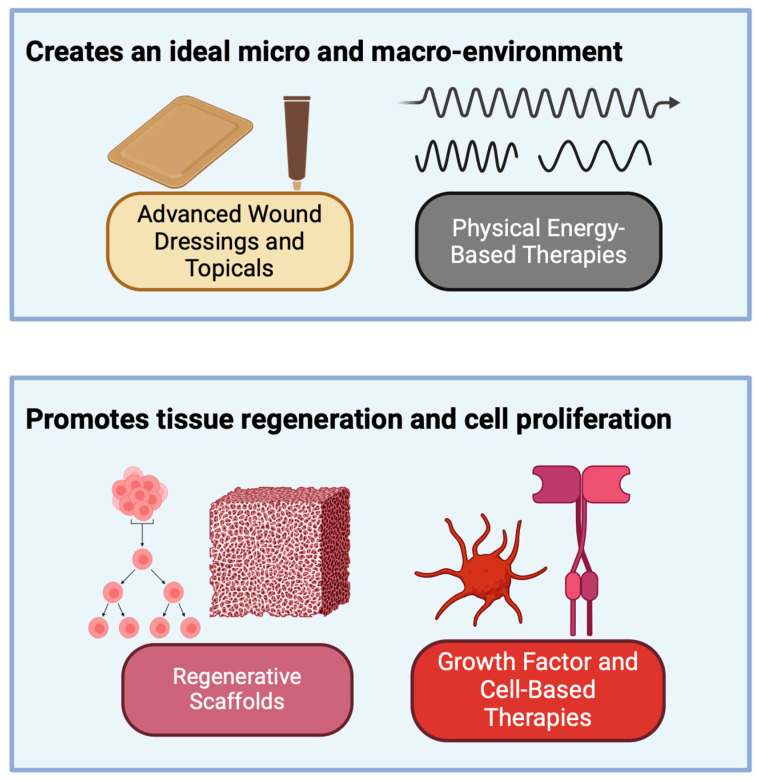
Classification of recent diabetic foot ulcer treatments. Created with BioRender.com.

In total, 59 articles spanned a variety of promising therapies for DFUs. Their findings are presented via the following four categories: advanced wound dressings and topicals, physical energy-based therapies, regenerative scaffolds, and growth factor- and cell-based therapies (Figure 4).

### 3.1. Advanced Wound Dressings and Topical Treatments

Dressings, topical ointments and gels, and topical applications of gaseous substances all work to create an ideal micro- and macroenvironment for the wound. They do this by means of physical control by absorbing exudate and managing moisture around the wound and infection control by preventing new bacteria from invading and via promoting oxygenation to the wound. Increased oxygenation stimulates immune system responses, supports the migration of digestive immune cells, disables toxins, and reduces inflammation. Although dressings, topical ointments, and topical gaseous applications facilitate wound healing by both methods of physical and infection control, they have been categorized below via their major mechanism of action.

#### 3.1.1. Physical Control

##### Absorption of Exudate

Therapies that utilize means of physical control include those that work to create an ideal microenvironment for healing by acting externally to protect the wound from the external environment. Dressings are an example of this therapy, as they are applied directly to the wound to promote healing, protect the area from contamination, absorb exudate, and manage moisture around the wound. Traditional dressings include gauze and foam, but many have explored the enhancement of these dressings, such as with antimicrobials, to better facilitate healing.

For example, silver-based and antimicrobial-based dressings have been explored for their efficacy in improving DFU healing. In a randomized trial, Lafontaine et al. studied silver dressings (Acticoat™, Smith & Nephew Medical Ltd., Durban, South Africa) versus non-silver dressings and found no significant difference in the proportion of ulcers healed by 12 weeks (n = 55, 75% vs. 69%, *p* = 0.49), suggesting that silver dressings did not offer any advantage over non-silver alternatives in healing outcomes or reducing antibiotic needs [4]. In contrast, Essa et al. evaluated SilvrSTAT Gel (SilvrStat™, ABL Medical, LLC, American Fork, UT, USA), a silver nanoparticle-based dressing, and found significantly better results, with 90% of ulcers completely healed by 12 weeks compared to 75% in the control group, suggesting that silver nanoparticles may accelerate healing and enhance the rate of wound closure [5]. Carbon is another material that has shown antimicrobial, absorbent, and adsorbent properties that facilitate wound healing. Bajuri et al. compared the Zorflex activated carbon cloth dressing to silver-based dressings and found that activated carbon cloth dressings resulted in a higher percentage of wound area reduction (85% vs. 65%) and more complete healing, indicating greater effectiveness [6]. Similarly, zinc oxide is known for its antimicrobial and anti-inflammatory properties, ability to promote fibroblast migration and biocompatibility. In a randomized-controlled trial, Loera-Valencia et al. investigated calcium alginate dressings with zinc oxide nanoparticles and found that the nanoparticle-enhanced dressings led to faster wound closure (75% vs. 71%, *p* = 0.01) and shorter healing times (48 days vs. 72 days), suggesting that nanoparticles improved tissue regeneration and healing speed [7]. Exploiting the same antimicrobial properties, Lullove studied polyvinyl alcohol foam dressings containing gentian violet/methylene blue and observed a 53% reduction in wound size after four weeks, with the foam’s antibacterial properties aiding in healing DFUs [8]. These findings highlight the potential of enhanced dressings and nanotechnology in improving DFU treatment outcomes.

##### Moist Wound Healing

Topical creams, ointments and gels, like dressings, can similarly physically protect a wound from external stressors, but they are better at creating a controlled, moist environment that facilitates healing. For example, Barbosa et al. compared autolytic debridement with a hydrogel enriched with sodium alginate and vitamins A and E to simple cleaning and dressing changes [9]. No statistically significant differences were found in wound healing or lesion area between the two groups, although the hydrogel group showed a reduction in inflammatory infiltrate without a corresponding increase in collagen production [9].

Similarly, Sahin et al. evaluated sodium pentaborate 3% gel’s moisturizing, antimicrobial, anti-inflammatory, and anti-oxidative abilities, along with its potential for improved angiogenesis and found that it significantly reduced ulcer grade and recurrence [10]. Similarly, Teobaldi I et al. examined a gel based on adelmidrol and trans-traumatic acid, which led to significant reductions in wound area and improved ulcer appearance, promoting re-epithelialization more effectively than standard care [11]. In another study, Meimeti et al. explored the use of an ointment containing olive oil extract from the marine isopod *Ceratothoa oestroides* and observed significant improvements in wound area reduction, skin hydration, and transepidermal water loss (*p* < 0.001), with 61% of patients experiencing complete ulcer healing using the extract alone [12].

Some gels are made more advanced, depending on their formulation, and they carry additional benefits, such as stimulating metabolism. Saghafi et al. compared liothyronine (T3), known for its metabolic enhancements, and liothyronine-insulin (T3/Ins) with honey cream and found that both T3 and T3/Ins significantly reduced healing time (15.9 days for T3, 16.4 days for T3/Ins vs. 60.6 days for the control), with all patients in the T3 and T3/Ins groups achieving full wound closure (*p* < 0.001) [13]. Gallelli et al. introduced a nano-hydrogel embedded with quercetin and oleic acid, which also significantly shortened healing times compared to hyaluronic acid, indicating its promise as an advanced treatment for DFUs [14]. Huang et al. investigated topical erythropoietin, which showed higher rates of partial wound closure (6 out of 10 patients) compared to the standard care group (1 out of 8 patients) [15]. Erythropoietin treatment resulted in faster wound area reduction (1.2 cm^2^ vs. 4.2 cm^2^, *p* = 0.02) and faster re-epithelialization [15].

Chitosan-based gels have shown promising results as well. Slivnik et al. demonstrated the efficacy of a chitosan-based gel compared to a placebo gel in improving wound closure [16]. By week ten, the chitosan-treated group achieved a 16.7% wound closure rate, significantly higher than the 4.2% closure observed in the placebo group, suggesting the gel’s potential to accelerate healing in DFUs [16].

These varied topical approaches demonstrate the potential of advanced gels, ointments, and innovative compounds in promoting faster healing and improved outcomes for DFU patients.

#### 3.1.2. Infection Control

Atmospheric therapies, including ozone, oxygen, and carbon dioxide, have been explored in DFU treatment for their antimicrobial and oxygenation abilities, with mixed results. Ozone has antimicrobial properties and can stimulate oxygenation to a wound site. Oxygen therapy also allows for increased oxygenation to a wound, enhancing cellular metabolism, tissue repair, and immunity. Carbon dioxide causes local vasodilation and a temporary acidic environment, prompting increased oxygenation to the area. Cold atmospheric plasma (CAP) promotes angiogenesis and enhanced oxygenation, stimulates cellular activity, and promotes immune activity.

Kadir et al. found that while ozone therapy significantly reduced bacterial colonization in DFUs, it did not result in statistically significant improvements in wound healing or assessment scores [17]. Similarly, Jonker et al. studied the use of Granulox (Granulox™, Hälsa Pharma GmbH, Lübeck, Germany), a hemoglobin spray designed to deliver oxygen to the wound surface, but found no significant difference in wound size reduction or healing rates compared to standard care [18].

In contrast, oxygen therapies have shown more promising outcomes. Lavery et al. found that continuous diffusion of oxygen significantly improved healing rates in grade 1A and 2A DFUs, with 46.2% of ulcers healed compared to 22.6% in the control group (*p* = 0.016) and even better results when frequent debridement was used [19]. He et al. further demonstrated that combining continuous diffusion of oxygen with traditional moist wound dressings led to higher healing rates and better infection control compared to either treatment alone [20]. In a randomized clinical trial with 145 patients, Serena et al. also observed improved outcomes with topical oxygen therapy, showing significantly higher wound closure rates (44.4% vs. 28.1%, *p* = 0.044) and greater ulcer size reduction (70% vs. 40%, *p* = 0.005) compared to standard care [21]. In a randomized trial with 220 patients, Frykberg et al. added to the evidence supporting oxygen-based treatments by assessing topical wound oxygen therapy, which significantly improved healing rates at both 12 weeks (42% vs. 14%, *p* = 0.01) and 12 months (56% vs. 27%, *p* = 0.013) [22].

Macura et al. investigated transcutaneous carbon dioxide application alongside standard wound care and found that carbon dioxide therapy resulted in significantly faster wound healing, with a 96% reduction in surface area and a 99% reduction in volume compared to the control group [23].

CAP therapy has also been explored as an adjunct to standard DFU treatment, showing promising results. Stratmann et al. tested CAP therapy compared to standard care and found that it significantly reduced wound area (mean area reduction: −26.31 units, *p* = 0.03) and accelerated wound reduction (10% reduction in 1.60 weeks, *p* = 0.009) [24]. However, CAP did not show a significant difference in infection reduction or microbial load compared to standard care. Samsavar et al. found that CAP reduced wound size and exudate, with improvements in wound grading observed by the 6th week of treatment [25]. Mirpour et al. also demonstrated that CAP significantly reduced wound size, with 77.3% of CAP-treated wounds reaching a predetermined size by 3 weeks compared to 36.4% in the control group [26]. However, while CAP reduced bacterial load immediately after exposure, it did not maintain a long-term antiseptic effects [26].

These studies suggest that while ozone therapy may not be as effective, oxygen, carbon dioxide, and CAP treatments show promise in improving healing rates and reducing ulcer size in DFUs. These therapies show potential as effective antimicrobials, though their impacts on long-term infection control requires further investigation.

### 3.2. Physical Energy-Based Therapies

#### 3.2.1. Negative Pressure Wound Therapy

Negative pressure wound therapy (NPWT) is a well-studied therapy that has gained recognition for its efficacy in promoting wound healing, particularly in chronic, hard-to-heal wounds like DFUs. NPWT, also known as vacuum-assisted closure (VAC), helps heal DFUs by using controlled, localized negative pressure or suction to promote wound healing. The suction removes excess fluid, dead tissue, and bacteria to promote wound drainage, prevent maceration, and reduce edema. Suction also stimulates microcirculation, which increases blood flow to the wound, bringing nutrients and oxygen to facilitate repair. NPWT also contributes to mechanical forces that pull together the wound edges and stimulate the formation of granulation tissue, vasculature, and collagen to fill the wound and promote contracture.

Several studies have explored the effectiveness of NPWT compared to other methods for treating DFUs, with mostly encouraging results. Seidel et al. found no significant difference in wound closure rate or time to closure when comparing NPWT to standard moist wound care (SMWC) over 6 months in a single-clinic study, but in a multicenter study, found that NPWT resulted in shorter treatment durations [27,28]. In a prospective randomized study, Maranna et al. reported more promising results, with NPWT leading to an early reduction in ulcer size, increased granulation tissue, shorter hospital stays, and more complete healing after three months compared to saline dressings [29]. Similarly, Wu et al. compared NPWT to alginate dressings for wound bed preparation before skin grafting and found that NPWT led to faster surgeries, higher graft survival rates, better wound perfusion and reduced formation of neutrophil extracellular traps [30].

Efforts to enhance NPWT have also shown potential. Campitiello et al. introduced a technique called “NPWT+”, in which foams are precisely shaped to fit the wound and an additional foam is wrapped around the foot [31]. They found that NPWT+ significantly reduced wound closure times and accelerated healing in DFUs [31]. However, in a randomized control trial with 150 patients, Lavery et al. found no significant differences in wound healing, infection rates, or hospital stays between NPWT with and without polyhexanide-betaine irrigation, suggesting that additional irrigation may not enhance NPWT’s effectiveness [32].

When comparing different types of NPWT, Kirsner et al. discovered that single-use NPWT had significantly higher wound closure rates after 12 weeks compared to traditional NPWT [33]. This finding suggests that, because of its disposable nature, single-use NPWT could be more affordable for less complex wounds.

Overall, while NPWT is well-studied as an important adjunct treatment for DFUs and offers advantages in specific contexts, especially for wound bed preparation and in resource-limited settings, the findings highlight the need for tailored approaches to maximize its benefits.

#### 3.2.2. Waveform-Based Therapies

Therapies relying on waveforms from physical energies include those leveraging mechanical, light, and electrical properties to enhance wound healing. Extracorporeal shockwave therapy (ESWT), photobiomodulation (PBM), and combined ultrasound and electrostimulation (CUSECS) are examples of these therapies. ESWT applies acoustic shockwaves to the wound area to generate mechanical and biological effects that stimulate healing. These effects include an increased blood flow, cellular activation, and anti-inflammatory properties. PBM uses light in the red or near-infrared spectrum to stimulate cellular processes and enhance cellular metabolism. CUSECS combines ultrasound waves with electrical stimulation to generate mechanical vibration and low electrical currents to activate cell metabolism.

The literature on physical energy therapies for DFUs Is growing but remains somewhat incomplete. ESWT, PBM, and CUSECS have shown potential in improving healing outcomes, though further research is needed to fully establish their effectiveness before widespread clinical application.

Vangaveti et al. investigated ESWT combined with standard of care (SoC) in a randomized trial involving 48 patients [34]. After 6 weeks of treatment, more patients in the ESWT + SoC group had healed DFUs compared to the SoC group, though the difference was not statistically significant [34]. The study suggests that while ESWT may offer some benefits for wound healing, larger trials with extended treatment periods are needed to substantiate its effectiveness.

Haze et al. evaluated the use of an at-home photobiomodulation (PBM) device for grade three DFUs in a frail population with significant comorbidities [35]. Over a 12-week period, the PBM-treated group experienced a significantly greater reduction in wound size compared to the placebo group (*p* = 0.018), with wound closure (>90% of the initial wound size) occurring in seven out of ten patients compared to only one out of ten in the sham group (*p* = 0.006) [35]. This suggests that PBM may be an effective home-based therapy for severe DFUs when combined with standard care.

Similarly, in a randomized controlled trial with 80 patients, Saura Cardoso et al. explored the impact of photobiomodulation on non-infected DFUs, using three different energy densities [36]. All PBM treatment groups saw significant ulcer size reductions (*p* < 0.05), with the highest healing rate observed in the group with the highest energy density [36]. This finding indicates that PBM, particularly at higher energy densities, can be highly effective in promoting long-term ulcer healing.

Taha et al. examined Bioptron light therapy, a type of PBM, for 40 Wagner grade 1 or 2 DFUs [37]. Over 2 months, the light therapy group experienced a significantly greater reduction in ulcer size (51% vs. 25%, *p* < 0.001) and a higher rate of bacterial clearance (60% vs. 15%, *p* = 0.02) compared to standard wound care alone [37]. Bioptron light therapy thus appears to be a promising adjunct to standard care in accelerating healing and reducing infection rates in DFUs.

Lastly, Hearne et al. studied the effectiveness of CUSECS in treating DFUs. The experimental group receiving CUSECS and standard care showed a higher rate of wound healing (mean difference = 0.49) compared to the control group, with two patients achieving full healing in the experimental group versus one in the control group [38]. However, due to the small sample size, the results were not statistically significant [38]. This study highlights the potential of CUSECS as a promising adjunct therapy, though larger trials are needed to confirm its effectiveness.

These studies suggest that newer therapies like ESWT, PBM, and CUSECS may offer significant benefits in the treatment of DFUs, though more extensive and larger-scale research is required to definitively establish their clinical efficacy.

### 3.3. Regenerative Scaffolds

#### 3.3.1. Dermal Replacement

Dermal replacement therapies rely on their abilities to provide a scaffold to support cell migration and angiogenesis and stimulate collagen to facilitate wound healing. These therapies have shown promising results in healing DFUs.

Kesavan et al. utilized a minimally manipulated extracellular matrix (MA-ECM) derived from autologous adipose tissue applied using 3D bioprinting, achieving complete wound closure within 4 weeks in the test group, while the control group experienced delayed healing [39]. Armstrong et al. compared a resorbable glass microfiber matrix (BBGFM) to SoC for DFUs and found that 70% of BBGFM-treated ulcers healed at 12 weeks compared to only 25% in the SoC group (*p* = 0.006) [40]. The BBGFM significantly improved both wound healing and neuropathic scores [40]. In another study, Armstrong et al. assessed the effectiveness of a purified reconstituted bilayer membrane (PRBM) for DFUs [41]. At 12 weeks, 85% of PRBM-treated wounds had closed compared to 30% of wounds treated with SoC (*p* = 0.0004), showing that PRBM not only improved healing time but also reduced complications associated with DFUs [41].

Hahn et al. combined micronized dermal matrix with NPWT for 30 Wagner grade 2 DFUs, resulting in 87% wound closure in the experimental group compared to 57% in the NPWT-only group (*p* = 0.04). This finding further demonstrates the matrix’s effectiveness in enhancing healing rates [42]. In a collagen-based study, Park et al. found that ulcers in 30 patients treated with porcine type I collagen had significantly higher complete healing rates (82% vs. 39%, *p* = 0.02), faster healing velocity, and shorter time to 50% size reduction compared to foam dressings alone [43].

These findings collectively emphasize the effectiveness of matrices and collagen-based therapies in enhancing wound healing, reducing ulcer size and promoting recovery in DFU patients.

#### 3.3.2. Stem Cell and Regenerative Approaches

Stem cell and regenerative approaches are known for their abilities to promote cellular regeneration and differentiation, enhance angiogenesis and anti-inflammation, secrete a wide array of bioactive molecules, produce extracellular matrix, and modulate the wound environment to contribute to a favorable microenvironment for DFU healing.

Stem cell and mesenchymal stromal cell (MSC) therapies hold significant promise for DFU treatment. Carstens et al. investigated the safety and efficacy of adipose-derived mesenchymal stromal cell (SVF) therapy in 63 patients with non-healing DFUs [44]. The results were compelling. Specifically, 81% of patients achieved full ulcer closure at 6 months, and this increased to 100% by 12 months [44]. Furthermore, Doppler studies indicated improved vascular function, highlighting not only the effectiveness of SVF therapy for wound healing but also its role in vascular repair [44].

Similarly, Arango-Rodriguez et al. examined the use of allogeneic human bone marrow mesenchymal stem cells (allo-hBM-MSCs) compared to PolyMem (PolyMem™, Ferris Mfg. Corp., Fort Worth, TX, USA) dressings in patients with Wagner grade 1 and 2 DFUs [45]. The results showed that allo-hBM-MSC-treated wounds exhibited greater closure, enhanced skin regeneration, and faster healing than the control group [45]. No adverse events were reported and proteomics analysis underscored the role of mesenchymal stromal cell-secreted factors in promoting effective wound healing [45].

In a related study, Zhang et al. explored the potential of human umbilical cord stem cells for treating refractory DFUs [46]. The therapy facilitated wound healing by enhancing angiogenesis and modulating immune responses, demonstrating the potential of umbilical cord stem cells in advanced wound care. Zhang et al. concluded that additional clinical trials are warranted to further investigate the therapeutic benefits of this stem cell-based approach [46].

These studies collectively suggest that cell-based regenerative therapies, including MSCs from adipose tissue, bone marrow, and umbilical cord stem cells, may revolutionize DFU treatment by promoting faster healing, improved tissue regeneration, and enhanced vascular repair.

#### 3.3.3. Amniotic-Based Therapies

Amniotic-based therapies have demonstrated promise in facilitating DFU healing due to their richness in growth factors and cytokines, anti-inflammatory effects, antimicrobial properties, reduced immunogenicity, and ability to serve as a natural scaffold for tissue regeneration.

Several studies have demonstrated the benefit of amniotic-based therapies. Cazzel et al. compared dehydrated amnion chorion membrane (dACM) to SoC in complex DFU cases and found significantly higher wound closure rates in the dACM group (50% vs. 35% by week 12, *p* = 0.04), alongside faster healing times [47]. Similarly, Game et al. evaluated the use of dried human amniotic membrane (dHAM) and observed a notable difference in healing rates, with 27% of ulcers in the dHAM group achieving full closure compared to just 6.3% in the SoC group (*p* = 0.0057) [48].

Serena et al. further supported these findings by examining hypothermically stored amniotic membrane [49]. Their results revealed significantly higher wound closure rates in the amniotic membrane group (60%) compared to the SoC group (37%, *p* = 0.04) [49].

Additionally, Niami et al. explored the impact of an amniotic fluid (AF) formulation on DFUs [50]. The study showed statistically significant improvements in wound healing, including wound grade, color, and the condition of surrounding tissue [50]. By the end of the eighth week, there was a notable difference in wound healing parameters among the four groups, with the AF gel demonstrating superior results compared to plain gauze [50].

Together, these studies highlight the potential of amniotic-based treatments to accelerate healing and improve outcomes in DFU patients.

### 3.4. Growth Factor- and Cell-Based Therapies

The research on platelet-rich plasma (PRP) has increased in recent years due to its suspected abilities to modulate inflammation, enhance collagen production, and release growth factors essential for wound healing. The literature on the topic remains mixed, however, as despite PRP’s theoretical benefits, the specifics of its execution have not been perfected.

In a small study, Alhawari et al. compared autologous PRP to platelet-poor plasma (PPP) and found that wound healing was achieved in nine out of ten patients in the PRP group, but in none of the patients in the PPP group by day 84 [51]. In contrast, in a study of 60 patients, Gupta et al. found no significant difference between PRP dressings and normal saline dressings in terms of healing rate or percentage ulcer area reduction [52]. Both groups showed similar reductions in ulcer area over 6 weeks (85.98% vs. 81.72%, *p* = 0.29), suggesting that in some cases, PRP may not provide added benefit over simpler treatments [52].

Other studies focused on PRP gel applications. Malekpour et al. found that PRP gel applied twice weekly significantly increased the healing rate of DFUs in 90 patients compared to conventional silver sulfadiazine ointment (*p* = 0.0), although PRP did not impact the need for amputations or other treatments (*p* = 0.11) [53]. Elsaid et al. also reported that PRP gel dressings led to a significant reduction in wound size (43.2% vs. 4.1%, *p* < 0.0001) and faster healing times (6.3 vs. 10.4 weeks, *p* < 0.0001) in 24 patients compared to saline dressings [54]. In a similar vein, Xie et al. used autologous platelet-rich gel and observed a higher sinus tract closure rate within the first 4 weeks, along with significantly shorter hospital stays (19 days vs. 48 days, *p* < 0.05) and lower total hospitalization costs in the autologous platelet-rich gel group [55].

Mohammadi et al. took a different approach by comparing dehydrated amnion dressings, platelet-derived growth factor gel, and surgical debridement in 243 patients with Wagner’s grades 1 and 2 DFUs [56]. The dehydrated amnion dressing proved superior, with an 86.4% reduction in wound area by week 8, compared to 43.7% in the debridement group and 50% in the growth factor group (*p* < 0.05) [56]. Hossam et al. explored PRP injections directly at the wound site and found that PRP led to faster healing, with a 50% reduction in ulcer surface area achieved earlier (2.5 weeks vs. 4.5 weeks, *p* < 0.001) [57]. PRP was also associated with significantly fewer wound infections (10% vs. 45%, *p* < 0.001) [57].

However, not all PRP studies demonstrated significant benefits, while some demonstrated benefit when combined with an additional compound. Smith et al. evaluated fat grafting with or without PRP compared to standard podiatric care for DFUs and found no significant differences in clinical outcomes, though the study suggested that a larger trial might be needed [58]. In a randomized controlled trial with 25 patients, Yarahmadi et al. assessed PRP-fibrin glue (FG) dressings combined with oral vitamins E and A versus PRP-FG with a placebo [59]. While both groups experienced significant wound size reductions, the vitamin-treated group saw greater improvements (*p* = 0.019) and reductions in oxidative stress markers (*p* < 0.05), indicating potential benefits of combining PRP with antioxidant therapy [59]. Kartika et al. compared autologous platelet-rich fibrin (A-PRF) plus hyaluronic acid (HA), A-PRF alone, and sodium chloride for DFU treatment [60]. The A-PRF + HA group showed significantly better angiogenesis and reduced inflammation, leading to improved wound healing outcomes compared to the other groups [60].

Several studies have explored the use of isolated concentrations of growth factors alone, which have also showed promising results in accelerating healing and improving tissue quality. Oliveira et al. investigated human recombinant epidermal growth factor (h-EGF) in 25 patients with DFUs and found that the h-EGF group had significantly greater wound area reduction (*p* = 0.049) and enhanced tissue quality compared to the control group, with no adverse events reported [61]. Similarly, Viswanathan et al. tested hEGF and demonstrated that 78% of ulcers in the hEGF group healed, compared to 52% in the placebo group [62]. The study also noted that hEGF significantly reduced healing time and boosted collagen and fibroblast development (*p* < 0.0001), highlighting its effectiveness in DFU treatment [62].

In summary, while growth factor-based therapies show promise in enhancing DFU healing, their efficacy varies depending on the method of application and the specific formulation used. Additionally, many studies involve only a small patient population, so more robust randomized trials must be performed (Table 1).

## 4. Discussion

The management of DFUs remains a critical challenge, particularly due to their complex pathophysiology contributing to the nonhealing nature of these wounds. The past five years have seen incredible advancements in DFU treatment, with novel therapies showing promise in improving outcomes.

For example, enhancing dressings and investigating ointments with antimicrobial agents or nanoparticles have shown improvements in wound area reduction and healing times, especially compared to traditional wound care including silver dressings and topical hydrogel. These advancements are particularly important in light of emerging silver-resistance, which necessitates alternative materials that can support wound healing and fight infection [63]. To avoid such resistance and improve upon existing dressings, further studies should compare different combinations of dressings and ointments to clarify their role in reducing infection and promoting healing. Advanced nanoparticle dressings, for example, represent a major step forward, as they offer targeted antimicrobial activity and enhance tissue regeneration. Providers should be made aware that they can enhance their usage of gauze dressings or hydrogel ointments with other, adjunctive methods that improve care.

Wound care staff should also be made aware of the benefits of NPWT, as it has become a common treatment modality for large DFUs, especially in preparing wounds beds for further interventions like skin grafts. While some studies showed its superiority over saline dressings or other standard methods, other research indicated a variability in outcomes. This variability underscores the importance of tailoring NPWT to individual patient needs, especially in terms of wound size, depth, and comorbidities. For example, single-use NPWT has shown promise in resource-limited settings due to its cost-effectiveness, whereas more advanced therapies, like NPWT+, may be better suited for severe cases. Future work should focus on defining when to employ these therapies to maximize clinical benefits.

Most atmospheric studies have shown to improve wound healing. In particular, the literature seems to agree that oxygen therapy, via continuous diffusion or topical application, improves DFU healing. Oxygen’s abilities to stimulate an immune response, angiogenesis, and a reduction in inflammation are central to treating DFUs. However, varied evidence for ozone and CAP highlights the need to standardize treatment protocols and conduct larger trials to confirm therapeutic efficacy. Clinicians should weight the relative benefits of these therapies, especially when considering DFUs with high bacterial content or low perfusion.

Similarly, despite initial results being very promising, larger, randomized trials should be done to confirm the efficacy and proper application of PRP, growth factor-based therapies, ESWT, PBM, and CUSECS before widespread application of these interventions. For example, growth factor therapies have shown potential in improving rates of wound closure. As cell-free therapies, they reduce risks of immunogenicity and tumorigenicity while being easier to transport and store. They offer regenerative benefits including reduced inflammation and enhanced angiogenesis. However, inconsistencies in dosing and delivery methods have posed challenges for widescale clinical implementation. Addressing these gaps could make these therapies more effective and accessible.

Matrix and collagen-based therapies, amniotic-based therapies, and regenerative approaches have been groundbreaking in enhancing healing through improvements in wound closure rates and frequency, especially for complex DFUs. Amniotic-based therapies, in particular, offer a unique combination of anti-inflammatory, antimicrobial, and regenerative properties that position them as a valuable option for severe DFUs. However, cost and accessibility remain as barriers, and reducing production cost could stand to facilitate broader usage. Further comparative studies between different matrices could help identify the ideal treatment for specific wound types, improving outcomes while minimizing resource use.

Moving forward, the field of DFU care needs a multi-faceted approach that enhances standard care with novel therapeutics. Providers will need to weigh the trade-offs of new technologies without robust clinical studies in deciding how to best apply these modalities to specific patients.

A few critical components of this multi-faceted approach include achieving optimal glycemic and pain control. Variability in glucose levels and chronic hyperglycemia are well-established as contributors to impaired wound healing. Contemporary strategies, including continuous glucose monitoring, individualized insulin regimens, and automated insulin delivery systems, all support a stable blood glucose. Additionally, addressing pain control is essential, as chronic pain can reduce patient adherence to care and affect their quality of life. Incorporating pain management strategies, such as analgesic use or nerve block therapies, along with advanced wound care, can optimize physical and psychological comfort and outcomes. These multi-faceted approaches can support wound healing.

By integrating insights from recent advancements (Table 2), in addition to known techniques, clinicians can begin to tailor treatments more precisely, considering factors like patient comorbidities, wound severity, and available resources. Additionally, a focus on personalized medicine, such as leveraging genetic markers or predictive analytics, could further optimize outcomes.

Future research should focus on larger patient cohorts in randomized prospective clinical trials supplemented by registry and retrospective studies to better inform clinicians about the specific patients that will benefit from each modality. Further research should also focus on making these therapies cost-effective and accessible, while possibly exploring additional routes, including personalized approaches that tailor treatments to specific patient needs based on their wound severity, comorbidities, and genetic factors. These efforts will be critical in addressing the persistent burden of DFUs, ultimately improving outcomes and reducing healthcare costs.

## Figures and Tables

**Table 1:** Characteristics of studies focused on platelet-rich plasma therapies.

Study	Total Number of Patients	Type of Study	Intervention and Comparison	Outcomes
Alhawari et al. [51]	10	Prospective clinical trial	Autologous PRP injections vs. platelet-poor plasma	Improved wound healing (*p* < 0.05) *
Gupta et al. [52]	60	Randomized controlled trial	PRP dressings vs. normal saline dressings	Healing rate or percentage ulcer area reduction (*p* = 0.3)
Malekpour et al. [53]	90	Randomized clinical trial	PRP gel vs. silver sulfadiazine ointment	Increased the healing rate (*p* = 0.0) *
Elsaid et al. [54]	24	Randomized clinical trial	PRP gel dressings vs. saline dressings	Reduced wound size and contributed to faster healing times (*p* < 0.00) *
Xie et al. [55]	48	Clinical study	Autologous platelet-rich gel vs. standard wound care	Shorter hospital stays (*p* < 0.05) *
Mohammadi et al. [56]	243	Randomized clinical trial	Dehydrated amnion dressings vs. platelet-derived growth factor gel vs. surgical debridement	Dehydrated amnion resulted in a reduction in wound area (*p* < 0.05) *
Hossam et al. [57]	80	Randomized clinical trial	PRP injections vs. standard wound care	Reduction in ulcer area (*p* <0.00) *
Smith et al. [58]	18	Feasibility-randomized controlled trial	Fat grafting with or without PRP vs. standard podiatric care	No significant differences
Yarahmadi et al. [59]	25	Double-blind, parallel-group clinical trial	PRP-fibrin glue dressings combined with oral vitamins E and A vs PRP-fibrin glue with a placebo	Improved wound healing (*p* = 0.019) *
Kartika et al. [60]	30	Open label, randomized controlled trial	Autologous platelet-rich fibrin (A-PRF) plus hyaluronic acid (HA) vs. A-PRF alone vs. sodium chloride	A-PRF + HA significantly improved wound healing (*p* < 0.05) *

* Significant difference.

**Table 2:** Comparison among recent therapy types.

Select Characteristics of Recent Therapies for DFUs				
Type of Therapy	Mechanism of Action	Select Advantages	Limitations	Practical Considerations
Advanced Wound Dressings and Topicals	Infection control, moisture balance, enhanced oxygenation	- Creates optimal microenvironment for healing - Enhances infection control with antimicrobial properties	- Resistance to certain agents (e.g., silver) - Mixed outcomes across dressing types	Widely available, easy to use, adaptable
Physical Energy-Based Therapies	Stimulates microcirculation, cellular activation, infection control	- Enhances granulation tissue formation - Improves cellular metabolism and oxygenation	- May require specialized equipment and training - Variable efficacy depending on wound type	Effective adjunct in severe cases
Regenerative Scaffolds	Supports cell migration and proliferation, stimulates angiogenesis	- Provides structural support for cellular regeneration - Encourages vascularization and collagen production	- Costly - Limited large-scale comparative studies	Suited for severe or complex wounds; resource-intensive
Growth Factor- and Cell-Based Therapies	Enhances collagen production, anti-inflammatory	- Modulates inflammation and promotes immune response - Enhances healing in chronic and hard-to-heal wounds	- Variability in dosing and delivery methods - Mixed evidence for certain therapies	Possible promise in advanced cases; preparation infrastructure may be required

## Data Availability

No new data were created or analyzed in this study.

## Supplementary Materials

The following supporting information can be downloaded at: https://www.mdpi.com/article/10.3390/jcm13247655/s1, Supplementary File S1: Lists screened studies.
