# Supplementary material for: An Overview of Recent Clinical Trials for Diabetic Foot Ulcer Therapies
Source: J Clin Med. 2024 Dec 16;13(24):7655. doi: 10.3390/jcm13247655 (PMC11676782; doi:10.3390/jcm13247655)
Supplement: Supplementary file 1 [file jcm-13-07655-s001.zip › jcm-3318790-supplementary.pdf]

Supplementary Document 1. List of all screened articles.

1. Armstrong DG, Orgill DP, Galiano RD, et al. A multi-centre, single-blinded randomised controlled clinical trial evaluating the effect of resorbable glass fibre matrix in the treatment of diabetic foot ulcers. Int Wound J. 2022;19(4):791-801. doi:10.1111/iwj.13675

2. Al-Jalodi O, Kupcella M, Breisinger K, Serena TE. A multicenter clinical trial evaluating the durability of diabetic foot ulcer healing in ulcers treated with topical oxygen and standard of care versus standard of care alone 1 year post healing. Int Wound J. 2022;19(7):1838-1842. doi:10.1111/iwj.13789

3. Lullove EJ, Liden B, Winters C, McEneaney P, Raphael A, Lantis Ii JC. A Multicenter, Blinded, Randomized Controlled Clinical Trial Evaluating the Effect of Omega-3-Rich Fish Skin in the Treatment of Chronic, Nonresponsive Diabetic Foot Ulcers. Wounds. 2021;33(7):169-177. doi:10.25270/wnds/2021.169177

4. Armstrong DG, Orgill DP, Galiano R, et al. A multicenter, randomized controlled clinical trial evaluating the effects of a novel autologous heterogeneous skin construct in the treatment of Wagner one diabetic foot ulcers: Final analysis. Int Wound J. 2023;20(10):4083-4096. doi:10.1111/iwj.14301

5. Frykberg RG, Franks PJ, Edmonds M, et al. A Multinational, Multicenter, Randomized, Double-Blinded, Placebo-Controlled Trial to Evaluate the Efficacy of Cyclical Topical Wound Oxygen (TWO2) Therapy in the Treatment of Chronic Diabetic Foot Ulcers: The TWO2 Study. Diabetes Care. 2020;43(3):616-624. doi:10.2337/dc19-0476

6. Rashid ST, Cavale N, Bowling FL. A pilot feasibility study of non-cultured autologous skin cell suspension for healing diabetic foot ulcers. Wound Repair Regen. 2020;28(6):719-727. doi:10.1111/wrr.12844

7. Jonker L, Smith D, Mark E, Thornthwaite S, Gunn C, Fisher S. A Pragmatic, Single-Center, Prospective, Randomized Controlled Trial of Adjunct Hemoglobin-Mediated Granulox Topical Oxygen Therapy Twice Weekly for Foot Ulcers. J Am Podiatr Med Assoc. 2021;111(5). doi:10.7547/19-189

8. Lullove E. A prospective, open-label, nonrandomized clinical trial using polyvinyl alcohol antibacterial foam for debridement of diabetic foot ulcers. Wounds. 2024;36(5):160-165. doi:10.25270/wnds/23008

9. Kirsner RS, Zimnitsky D, Robinson M. A prospective, randomized, controlled clinical study on the effectiveness of a single-use negative pressure wound therapy system, compared to traditional negative pressure wound therapy in the treatment of diabetic ulcers of the lower extremities. Wound Repair Regen. 2021;29(6):908-911. doi:10.1111/wrr.12966

10. Armstrong DG, Orgill DP, Galiano RD, et al. A purified reconstituted bilayer matrix shows improved outcomes in treatment of non-healing diabetic foot ulcers when compared to the standard of care: Final results and analysis of a prospective, randomized, controlled, multi-centre clinical trial. Int Wound J. 2024;21(4):e14882. doi:10.1111/iwj.14882

11. Serena TE, Yaakov R, Moore S, et al. A randomized controlled clinical trial of a hypothermically stored amniotic membrane for use in diabetic foot ulcers. J Comp Eff Res. 2020;9(1):23-34. doi:10.2217/cer-2019-0142

12. Nube VL, White JM, Brewer K, et al. A Randomized Trial Comparing Weekly With Every Second Week Sharp Debridement in People With Diabetes-Related Foot Ulcers Shows Similar Healing Outcomes: Potential Benefit to Resource Utilization. Diabetes Care. 2021;44(12):e203-e205. doi:10.2337/dc21-1454

13. Slivnik M, Navodnik Preložnik M, Fir M, et al. A randomized, placebo-controlled study of chitosan gel for the treatment of chronic diabetic foot ulcers (the CHITOWOUND study). BMJ Open Diabetes Res Care. 2024;12(3). doi:10.1136/bmjdrc-2024-004195

14. Bajuri MY, Nordin A. Activated carbon cloth versus silver-based dressings in a population with diabetic foot ulcer: a randomised controlled trial. J Wound Care. 2024;33(5):298-303. doi:10.12968/jowc.2024.33.5.298

15. Dos Santos CM, de Souza Lima da Silveira PR, da Rocha RB, et al. Adherence to Self-care and Quality of Life of Patients with Diabetic Foot Ulcers Treated with Low-Level Laser Therapy: An Exploratory Study. Adv Skin Wound Care. 2024;37(8):1-10. doi:10.1097/ASW.0000000000000189

16. Hossam EM, Alserr AHK, Antonopoulos CN, Zaki A, Eldaly W. Autologous Platelet Rich Plasma Promotes the Healing of Non-Ischemic Diabetic Foot Ulcers. A Randomized Controlled Trial. Ann Vasc Surg. 2022;82:165-171. doi:10.1016/j.avsg.2021.10.061

17. Xie J, Fang Y, Zhao Y, Cao D, Lv Y. Autologous Platelet-Rich Gel for the Treatment of Diabetic Sinus Tract Wounds: A Clinical Study. J Surg Res. 2020;247:271-279. doi:10.1016/j.jss.2019.09.069

18. Mirpour S, Fathollah S, Mansouri P, et al. Cold atmospheric plasma as an effective method to treat diabetic foot ulcers: A randomized clinical trial. Sci Rep. 2020;10(1):10440. doi:10.1038/s41598-020-67232-x

19. Park KH, Kwon JB, Park JH, Shin JC, Han SH, Lee JW. Collagen dressing in the treatment of diabetic foot ulcer: A prospective, randomized, placebo-controlled, single-center study. Diabetes Res Clin Pract. 2019;156:107861. doi:10.1016/j.diabres.2019.107861

20. Çetinkalp Ş, Gökçe EH, Şimşir I, et al. Comparative Evaluation of Clinical Efficacy and Safety of Collagen Laminin-Based Dermal Matrix Combined With Resveratrol Microparticles (Dermalix) and Standard Wound Care for Diabetic Foot Ulcers. Int J Low Extrem Wounds. 2021;20(3):217-226. doi:10.1177/1534734620907773

21. Essa MS, Ahmad KS, Zayed ME, Ibrahim SG. Comparative Study Between Silver Nanoparticles Dressing (SilvrSTAT Gel) and Conventional Dressing in Diabetic Foot Ulcer Healing: A Prospective Randomized Study. Int J Low Extrem Wounds. 2023;22(1):48-55. doi:10.1177/1534734620988217

22. Mohammadi Tofigh A, Tajik M. Comparing the standard surgical dressing with dehydrated amnion and platelet-derived growth factor dressings in the healing rate of diabetic foot ulcer: A randomized clinical trial. Diabetes Res Clin Pract. 2022;185:109775. doi:10.1016/j.diabres.2022.109775

23. Liao C, Zhu M, Ding H, Li Y, Sun Q, Li X. Comparing the traditional and emerging therapies for enhancing wound healing in diabetic patients: A pivotal examination. Int Wound J. 2024;21(3):e14488. doi:10.1111/iwj.14488

24. Probst S, Saini C, Skinner MB. Comparison of sterile polyacrylate wound dressing with activated carbon cloth and a standard non-adhesive hydrocellular foam dressing with silver: a randomised controlled trial protocol. J Wound Care. 2019;28(11):722-728. doi:10.12968/jowc.2019.28.11.722

25. Armstrong DG, Orgill DP, Galiano R, et al. Complete wound closure following a single topical application of a novel autologous homologous skin construct: first evaluation in an open-label, single-arm feasibility study in diabetic foot ulcers. Int Wound J. 2020;17(5):1366-1375. doi:10.1111/iwj.13404

26. Tong J, Zhang J, Xiang L, et al. Continuous intrafemoral artery infusion of urokinase improves diabetic foot ulcers healing and decreases cardiovascular events in a long-term follow-up study. BMJ Open Diabetes Res Care. 2024;12(1). doi:10.1136/bmjdrc-2023-003414

27. Cazzell SM, Caporusso J, Vayser D, Davis RD, Alvarez OM, Sabolinski ML. Dehydrated Amnion Chorion Membrane versus standard of care for diabetic foot ulcers: a randomised controlled trial. J Wound Care. 2024;33:S4-S14. doi:10.12968/jowc.2024.0139

28. Lavery LA, Niederauer MQ, Papas KK, Armstrong DG. Does Debridement Improve Clinical Outcomes in People With Diabetic Foot Ulcers Treated With Continuous Diffusion of Oxygen? Wounds. 2019;31(10):246-251.

29. Lavery LA, Davis KE, La Fontaine J, et al. Does negative pressure wound therapy with irrigation improve clinical outcomes? A randomized clinical trial in patients with diabetic foot infections. Am J Surg. 2020;220(4):1076-1082. doi:10.1016/j.amjsurg.2020.02.044

30. Saura Cardoso V, de Souza Lima da Silveira PR, Dos Santos CM, et al. Dose-response and efficacy of 904 nm photobiomodulation on diabetic foot ulcers healing: a randomized controlled trial. Lasers Med Sci. 2024;39(1):142. doi:10.1007/s10103-024-04090-3

31. Huang YY, Lin CW, Cheng NC, et al. Effect of a Novel Macrophage-Regulating Drug on Wound Healing in Patients With Diabetic Foot Ulcers: A Randomized Clinical Trial. JAMA Netw Open. 2021;4(9):e2122607. doi:10.1001/jamanetworkopen.2021.22607

32. Teobaldi I, Stoico V, Perrone F, et al. Effect of a Topical Gel Based on Adelmidrol + Trans-Traumatic Acid in the Treatment of Diabetic Foot Ulcers: An Open-Label Study. J Am Podiatr Med Assoc. 2022;112(1). doi:10.7547/20-143

33. Stratmann B, Costea TC, Nolte C, et al. Effect of Cold Atmospheric Plasma Therapy vs Standard Therapy Placebo on Wound Healing in Patients With Diabetic Foot Ulcers: A Randomized Clinical Trial. JAMA Netw Open. 2020;3(7):e2010411. doi:10.1001/jamanetworkopen.2020.10411

34. Jaber D, Younes N, Khalil E, et al. Effect of Diluted Dakin's Solution Versus Standard Care on Diabetic Foot Ulcer Management: A Randomized Controlled Trial. J Am Podiatr Med Assoc. 2022;112(1). doi:10.7547/20-213

35. Wang G, Li X, Ju S, et al. Effect of electrospun poly (L-lactide-co-caprolactone) and formulated porcine fibrinogen for diabetic foot ulcers. Eur J Pharm Sci. 2024;198:106800. doi:10.1016/j.ejps.2024.106800

36. Taha MM, El-Nagar MM, Elrefaey BH, et al. Effect of Polarized Light Therapy (Bioptron) on Wound Healing and Microbiota in Diabetic Foot Ulcer: A Randomized Controlled Trial. Photobiomodul Photomed Laser Surg. 2022;40(12):792-799. doi:10.1089/photob.2021.0175

37. Oliveira BC, de Oliveira BGRB, Deutsch G, Pessanha FS, de Castilho SR. Effectiveness of a synthetic human recombinant epidermal growth factor in diabetic patients wound healing: Pilot, double-blind, randomized clinical controlled trial. Wound Repair Regen. 2021;29(6):920-926. doi:10.1111/wrr.12969

38. Hearne CLJ, Patton D, Moore ZE, Wilson P, Gillen C, O'Connor T. Effectiveness of combined modulated ultrasound and electric current stimulation to treat diabetic foot ulcers. J Wound Care. 2022;31(1):12-20. doi:10.12968/jowc.2022.31.1.12

39. Sanpinit S, Chokpaisarn J, Na-Phatthalung P, et al. Effectiveness of Ya-Samarn-Phlae in diabetic wound healing: Evidence from in vitro studies and a multicenter randomized controlled clinical trial. J Ethnopharmacol. 2024;326:117929. doi:10.1016/j.jep.2024.117929

40. Snyder R, Nouvong A, Ulloa J, et al. Efficacy and safety of autologous whole blood clot in diabetic foot ulcers: a randomised controlled trial. J Wound Care. 2024;33(9):688-700. doi:10.12968/jowc.2024.0195

41. Liden BA, Ramirez-GarciaLuna JL. Efficacy of a polylactic acid matrix for the closure of Wagner grade 1 and 2 diabetic foot ulcers: a single-center, prospective randomized trial. Wounds. 2023;35(8):E257-E260. doi:10.25270/wnds/23094

42. Ohura N, Kimura C, Ando H, et al. Efficacy of autologous platelet-rich plasma gel in patients with hard-to-heal diabetic foot ulcers: a multicentre study in Japan. J Wound Care. 2024;33(7):484-494. doi:10.12968/jowc.2023.0088

43. Gupta A, Channaveera C, Sethi S, Ranga S, Anand V. Efficacy of Intralesional Platelet-Rich Plasma in Diabetic Foot Ulcer. J Am Podiatr Med Assoc. 2021;111(3). doi:10.7547/19-149

44. Miranda E, Bramono K, Yunir E, et al. Efficacy of LL-37 cream in enhancing healing of diabetic foot ulcer: a randomized double-blind controlled trial. Arch Dermatol Res. 2023;315(9):2623-2633. doi:10.1007/s00403-023-02657-8

45. Viswanathan V, Juttada U, Babu M. Efficacy of Recombinant Human Epidermal Growth Factor (Regen-D 150) in Healing Diabetic Foot Ulcers: A Hospital-Based Randomized Controlled Trial. Int J Low Extrem Wounds. 2020;19(2):158-164. doi:10.1177/1534734619892791

46. Saghafi F, Khalilzadeh SH, Ramezani V, Pasandeh F, Fallahzadeh H, Sahebnasagh A. Efficacy of the Novel Formulation of Topical Liothyronine and Liothyronine-insulin in Mild to Moderate Diabetic Foot Ulcer: A Randomized, Triple-blind Clinical Trial. Curr Med Chem. 2024;31(21):3232-3243. doi:10.2174/0929867330666230523155739

47. Niami F, Molavynejad S, Hemmati AA, et al. Evaluation of the effect of a gel made with amniotic fluid formulation on the healing of diabetic foot ulcers: A triple-blind clinical trial. Front Public Health. 2022;10:1025391. doi:10.3389/fpubh.2022.1025391

48. Malekpour Alamdari N, Shafiee A, Mirmohseni A, Besharat S. Evaluation of the efficacy of platelet-rich plasma on healing of clean diabetic foot ulcers: A randomized clinical trial in Tehran, Iran. Diabetes Metab Syndr. 2021;15(2):621-626. doi:10.1016/j.dsx.2021.03.005

49. Loera-Valencia R, Neira RE, Urbina BP, Camacho A, Galindo RB. Evaluation of the therapeutic efficacy of dressings with ZnO nanoparticles in the treatment of diabetic foot ulcers. Biomed Pharmacother. 2022;155:113708. doi:10.1016/j.biopha.2022.113708

50. Lázaro-Martínez JL, López-Moral M, García-Alamino JM, Bohbot S, Sanz-Corbalán I, García-Álvarez Y. Evolution of the TcPO(2) values following hyperoxygenated fatty acids emulsion application in patients with diabetic foot disease: results of a clinical trial. J Wound Care. 2021;30(1):74-79. doi:10.12968/jowc.2021.30.1.74

51. Campitiello F, Mancone M, Corte AD, Guerniero R, Canonico S. Expanded negative pressure wound therapy in healing diabetic foot ulcers: a prospective randomised study. J Wound Care. 2021;30(2):121-129. doi:10.12968/jowc.2021.30.2.121

52. Vangaveti VN, Jhamb S, Goodall J, Bulbrook J, Biros E, Malabu UH. Extracorporeal Shockwave Therapy (ESWT) in the Management of Diabetic Foot Ulcer: A Prospective Randomized Clinical Trial. J Foot Ankle Surg. 2023;62(5):845-849. doi:10.1053/j.jfas.2023.04.013

53. Smith OJ, Leigh R, Kanapathy M, et al. Fat grafting and platelet-rich plasma for the treatment of diabetic foot ulcers: A feasibility-randomised controlled trial. Int Wound J. 2020;17(6):1578-1594. doi:10.1111/iwj.13433

54. Lantis JC, Snyder R, Reyzelman AM, et al. Fetal bovine acellular dermal matrix for the closure of diabetic foot ulcers: a prospective randomised controlled trial. J Wound Care. 2021;30:S18-S27. doi:10.12968/jowc.2021.30.Sup7.S18

55. Lantis Ii JC, Lullove EJ, Liden B, et al. Final efficacy and cost analysis of a fish skin graft vs standard of care in the management of chronic diabetic foot ulcers: a prospective, multicenter, randomized controlled clinical trial. Wounds. 2023;35(4):71-79. doi:10.25270/wnds/22094

56. Barbosa MG, Carvalho VF, Paggiaro AO. Hydrogel enriched with sodium alginate and vitamins A and E for diabetic foot ulcer: a randomized controlled trial. Wounds. 2022;34(9):229-235. doi:10.25270/wnds/20103

57. Gould LJ, Orgill DP, Armstrong DG, et al. Improved healing of chronic diabetic foot wounds in a prospective randomised controlled multi-centre clinical trial with a microvascular tissue allograft. Int Wound J. 2022;19(4):811-825. doi:10.1111/iwj.13679

58. Halschou-Jensen PM, Sauer J, Bouchelouche P, Fabrin J, Brorson S, Ohrt-Nissen S. Improved Healing of Diabetic Foot Ulcers After High-dose Vitamin D: A Randomized Double-blinded Clinical Trial. Int J Low Extrem Wounds. 2023;22(3):466-474. doi:10.1177/15347346211020268

59. Dymarek R, Kuberka I, Walewicz K, Taradaj J, Rosińczuk J, Sopel M. Is Shock Wave Application Effective on Various Chronic Wounds in the Geriatric Population? Preliminary Clinical Study. Clin Interv Aging. 2024;19:665-679. doi:10.2147/CIA.S448298

60. Kesavan R, Sheela Sasikumar C, Narayanamurthy VB, Rajagopalan A, Kim J. Management of Diabetic Foot Ulcer with MA-ECM (Minimally Manipulated Autologous Extracellular Matrix) Using 3D Bioprinting Technology - An Innovative Approach. Int J Low Extrem Wounds. 2024;23(1):161-168. doi:10.1177/15347346211045625

61. Armstrong DG, Galiano RD, Orgill DP, et al. Multi-centre prospective randomised controlled clinical trial to evaluate a bioactive split thickness skin allograft vs standard of care in the treatment of diabetic foot ulcers. Int Wound J. 2022;19(4):932-944. doi:10.1111/iwj.13759

62. Liu Y, Li Y, Du Y, Huang T, Zhu C. Multicenter Clinical Trials Analyzing Efficacy and Safety of Topical Cortex Phellodendri Compound Fluid in Treatment of Diabetic Foot Ulcers. Med Sci Monit. 2020;26:e923424. doi:10.12659/MSM.923424

63. Gallelli G, Cione E, Serra R, et al. Nano-hydrogel embedded with quercetin and oleic acid as a new formulation in the treatment of diabetic foot ulcer: A pilot study. Int Wound J. 2020;17(2):485-490. doi:10.1111/iwj.13299

64. Wu Y, Shen G, Hao C. Negative pressure wound therapy (NPWT) is superior to conventional moist dressings in wound bed preparation for diabetic foot ulcers: A randomized controlled trial. Saudi Med J. 2023;44(10):1020-1029. doi:10.15537/smj.2023.44.20230386

65. Seidel D, Storck M, Lawall H, et al. Negative pressure wound therapy compared with standard moist wound care on diabetic foot ulcers in real-life clinical practice: results of the German DiaFu-RCT. BMJ Open. 2020;10(3):e026345. doi:10.1136/bmjopen-2018-026345

66. Maranna H, Lal P, Mishra A, et al. Negative pressure wound therapy in grade 1 and 2 diabetic foot ulcers: A randomized controlled study. Diabetes Metab Syndr. 2021;15(1):365-371. doi:10.1016/j.dsx.2021.01.014

67. Rastogi A, Kulkarni SA, Deshpande SK, et al. Novel Topical Esmolol Hydrochloride (Galnobax) for Diabetic Foot Wound: Phase 1/2, Multicenter, Randomized, Double-Blind,

Vehicle-Controlled Parallel-Group Study. Adv Wound Care (New Rochelle). 2023;12(8):429-439. doi:10.1089/wound.2022.0093

68. Seidel D, Lefering R. NPWT resource use compared with standard moist wound care in diabetic foot wounds: DiaFu randomized clinical trial results. J Foot Ankle Res. 2022;15(1):72. doi:10.1186/s13047-022-00569-w

69. Meimeti E, Tentolouris N, Manes C, et al. Ointments containing Ceratothoa oestroides extract: Evaluation of their healing potential in the treatment of diabetic foot ulcers. Wound Repair Regen. 2020;28(2):234-241. doi:10.1111/wrr.12771

70. Ajjan RA, Hensor EMA, Del Galdo F, et al. Oral 11β-HSD1 inhibitor AZD4017 improves wound healing and skin integrity in adults with type 2 diabetes mellitus: a pilot randomized controlled trial. Eur J Endocrinol. 2022;186(4):441-455. doi:10.1530/EJE-21-1197

71. Kadir K, Syam Y, Yusuf S, Zainuddin M. Ozone Therapy on Reduction of Bacterial Colonies and Acceleration of Diabetic Foot Ulcer Healing. Home Healthc Now. 2020;38(4):215-220. doi:10.1097/NHH.0000000000000889

72. Alhawari H, Jafar H, Al Soudi M, et al. Perilesional injections of human platelet lysate versus platelet poor plasma for the treatment of diabetic foot ulcers: A double-blinded prospective clinical trial. Int Wound J. 2023;20(8):3116-3122. doi:10.1111/iwj.14186

73. Wu Z, Li J, Li Z, Huang H, Shi Y, Li X. Plastic surgical repair of ulcer wounds of diabetic foot patients through systemic treatment and local infection control. Ann Palliat Med. 2022;11(4):1453-1461. doi:10.21037/apm-22-352

74. Lafontaine N, Jolley J, Kyi M, et al. Prospective randomised placebo-controlled trial assessing the efficacy of silver dressings to enhance healing of acute diabetes-related foot ulcers. Diabetologia. 2023;66(4):768-776. doi:10.1007/s00125-022-05855-7

75. Zhang J, Zhao B, Wei W, et al. Prospective, Randomized, and Controlled Study of a Human Umbilical Cord Mesenchymal Stem Cell Injection for Treating Diabetic Foot Ulcers. J Vis Exp. 2023;(193). doi:10.3791/65045

76. Elsaid A, El-Said M, Emile S, Youssef M, Khafagy W, Elshobaky A. Randomized Controlled Trial on Autologous Platelet-Rich Plasma Versus Saline Dressing in Treatment of Non-healing Diabetic Foot Ulcers. World J Surg. 2020;44(4):1294-1301. doi:10.1007/s00268-019-05316-0

77. Hahn HM, Lee DH, Lee IJ. Ready-to-Use Micronized Human Acellular Dermal Matrix to Accelerate Wound Healing in Diabetic Foot Ulcers: A Prospective Randomized Pilot Study. Adv Skin Wound Care. 2021;34(5):1-6. doi:10.1097/01.ASW.0000741512.57300.6d

78. Arango-Rodríguez ML, Solarte-David VA, Becerra-Bayona SM, et al. Role of mesenchymal stromal cells derivatives in diabetic foot ulcers: a controlled randomized phase 1/2 clinical trial. Cytotherapy. 2022;24(10):1035-1048. doi:10.1016/j.jcyt.2022.04.002

79. Shetty R, Giridhar BS, Potphode A. Role of ultrathin skin graft in early healing of diabetic foot ulcers: a randomized controlled trial in comparison with conventional methods. Wounds. 2022;33(2):57-67. doi:10.25270/wnds/2022.5767

80. Weenig RH, Davis MDP, Dahl PR, Su WPD. Skin Ulcers Misdiagnosed as Pyoderma Gangrenosum. N Engl J Med. 2002;347(18):1412-1418. doi:10.1056/NEJMoa013383

81. Irani PS, Ranjbar H, Mehdipour-Rabori R, Torkaman M, Amirsalari S, Alazmani-Noodeh F. The Effect of Aloe vera on the Healing of Diabetic Foot Ulcer: A Randomized, Double-blind Clinical Trial. Curr Drug Discov Technol. 2024;21(3):56-63. doi:10.2174/1570163820666230904150945

82. Kardoust M, Salehi H, Taghipour Z, Sayadi A. The Effect of Kiwifruit Therapeutics in the Treatment of Diabetic Foot Ulcer. Int J Low Extrem Wounds. 2021;20(2):104-110. doi:10.1177/1534734619851700

83. Yarahmadi A, Saeed Modaghegh MH, Mostafavi-Pour Z, et al. The effect of platelet-rich plasma-fibrin glue dressing in combination with oral vitamin E and C for treatment of non-healing diabetic foot ulcers: a randomized, double-blind, parallel-group, clinical trial. Expert Opin Biol Ther. 2021;21(5):687-696. doi:10.1080/14712598.2021.1897100

84. Şahin F, Pirouzpanah MB, Farshbaf-Khalili A, et al. The effect of the boron-based gel on the treatment of diabetic foot ulcers: A prospective, randomized controlled trial. J Trace Elem Med Biol. 2023;79:127261. doi:10.1016/j.jtemb.2023.127261

85. Abdoli A, Shahbazi R, Zoghi G, et al. The effect of topical olive oil dressing on the healing of grade 1 and 2 diabetic foot ulcers: An assessor-blind randomized controlled trial in type 2 diabetes patients. Diabetes Metab Syndr. 2022;16(12):102678. doi:10.1016/j.dsx.2022.102678

86. Macura M, Ban Frangez H, Cankar K, Finžgar M, Frangez I. The effect of transcutaneous application of gaseous CO(2) on diabetic chronic wound healing-A double-blind randomized clinical trial. Int Wound J. 2020;17(6):1607-1614. doi:10.1111/iwj.13436

87. Game F, Gray K, Davis D, et al. The effectiveness of a new dried human amnion derived membrane in addition to standard care in treating diabetic foot ulcers: A patient and assessor blind, randomised controlled pilot study. Int Wound J. 2021;18(5):692-700. doi:10.1111/iwj.13571

88. Bacelar de Assis B, de Cássia Lopes Chaves E, de Sousa L, et al. The effects of auricular acupuncture on vascular parameters on the risk factors for diabetic foot: A randomized clinical trial. Complement Ther Clin Pract. 2021;44:101442. doi:10.1016/j.ctcp.2021.101442

89. Mokhtari M, Razzaghi R, Momen-Heravi M. The effects of curcumin intake on wound healing and metabolic status in patients with diabetic foot ulcer: A randomized, double-blind, placebo-controlled trial. Phytother Res. 2021;35(4):2099-2107. doi:10.1002/ptr.6957

90. Du L, Zeng D, Hu X, Ren X, He D. The Efficacy of Autologous Platelet-Rich Gel and Traditional Chinese Medicine in Diabetic Foot Treatment: A Parallel Randomized Controlled Clinical Trial. Ann Vasc Surg. 2022;87:529-537. doi:10.1016/j.avsg.2022.07.026

91. Samsavar S, Mahmoudi H, Shakouri R, et al. The evaluation of efficacy of atmospheric pressure plasma in diabetic ulcers healing: A randomized clinical trial. Dermatol Ther. 2021;34(6):e15169. doi:10.1111/dth.15169

92. Dymarek R, Kuberka I, Rosińczuk J, Walewicz K, Taradaj J, Sopel M. The Immediate Clinical Effects Following a Single Radial Shock Wave Therapy in Pressure Ulcers: A Preliminary Randomized Controlled Trial of The SHOWN Project. Adv Wound Care (New Rochelle). 2023;12(8):440-452. doi:10.1089/wound.2021.0015

93. He S, Liang C, Yi C, Wu M. Therapeutic effect of continuous diffusion of oxygen therapy combined with traditional moist wound dressing therapy in the treatment of diabetic foot ulcers. Diabetes Res Clin Pract. 2021;174:108743. doi:10.1016/j.diabres.2021.108743

94. Fallah Huseini H, Yaghoobi M, Fallahi F, et al. Topical Administration of Teucrium polium on Diabetic Foot Ulcers Accelerates Healing: A Placebo-Controlled Randomized Clinical Study. Int J Low Extrem Wounds. 2024;23(2):238-246. doi:10.1177/15347346211048371

95. Hamed S, Ullmann Y, Belokopytov M, et al. Topical Erythropoietin Accelerates Wound Closure in Patients with Diabetic Foot Ulcers: A Prospective, Multicenter, Single-Blind, Randomized, Controlled Trial. Rejuvenation Res. 2021;24(4):251-261. doi:10.1089/rej.2020.2397

96. Rastogi A, Kulkarni SA, Agarwal S, et al. Topical Esmolol Hydrochloride as a Novel Treatment Modality for Diabetic Foot Ulcers: A Phase 3 Randomized Clinical Trial. JAMA Netw Open. 2023;6(5):e2311509. doi:10.1001/jamanetworkopen.2023.11509

97. Serena TE, Bullock NM, Cole W, et al. Topical oxygen therapy in the treatment of diabetic foot ulcers: a multicentre, open, randomised controlled clinical trial. J Wound Care. 2021;30:S7-S14. doi:10.12968/jowc.2021.30.Sup5.S7

98. Lipsky BA, Kim PJ, Murphy B, McKernan PA, Armstrong DG, Baker BHJ. Topical pravibismane as adjunctive therapy for moderate or severe diabetic foot infections: A phase 1b randomized, multicenter, double-blind, placebo-controlled trial. Int Wound J. 2024;21(4):e14817. doi:10.1111/iwj.14817

99. Kerstan A, Dieter K, Niebergall-Roth E, et al. Translational development of ABCB5(+) dermal mesenchymal stem cells for therapeutic induction of angiogenesis in non-healing diabetic foot ulcers. Stem Cell Res Ther. 2022;13(1):455. doi:10.1186/s13287-022-03156-9

100. Carstens MH, Quintana FJ, Calderwood ST, et al. Treatment of chronic diabetic foot ulcers with adipose-derived stromal vascular fraction cell injections: Safety and evidence of efficacy at 1 year. Stem Cells Transl Med. 2021;10(8):1138-1147. doi:10.1002/sctm.20-0497

101. Haze A, Gavish L, Elishoov O, et al. Treatment of diabetic foot ulcers in a frail population with severe co-morbidities using at-home photobiomodulation laser therapy: a double-blind, randomized, sham-controlled pilot clinical study. Lasers Med Sci. 2022;37(2):919-928. doi:10.1007/s10103-021-03335-9

102. Moeini S, Gottlieb H, Jørgensen TS, Larsen MRB, Brorson S. Treatment of Diabetic Foot Ulcers With Inforatio Technique to Promote Wound Healing: A Feasibility Trial. Int J Low Extrem Wounds. 2023;22(2):241-250. doi:10.1177/15347346211002364

103. Lee YJ, Han HJ, Shim HS. Treatment of hard-to-heal wounds in ischaemic lower extremities with a novel fish skin-derived matrix. J Wound Care. 2024;33(5):348-356. doi:10.12968/jowc.2024.33.5.348

104. Armstrong DG, Orgill DP, Galiano RD, et al. Use of a purified reconstituted bilayer matrix in the management of chronic diabetic foot ulcers improves patient outcomes vs standard of care: Results of a prospective randomised controlled multi-centre clinical trial. Int Wound J. 2022;19(5):1197-1209. doi:10.1111/iwj.13715

105. Lullove E. Use of fetal bovine dermal repair scaffold in diabetic foot ulcers with recidivism: an open-label prospective clinical study. J Wound Care. 2023;32:S10-S16. doi:10.12968/jowc.2023.32.Sup2.S10

106. Nik Hisamuddin NAR, Wan Mohd Zahiruddin WN, Mohd Yazid B, Rahmah S. Use of hyperbaric oxygen therapy (HBOT) in chronic diabetic wound - A randomised trial. Med J Malaysia. 2019;74(5):418-424.

107. Gunton JE, Girgis CM, Lau T, Vicaretti M, Begg L, Flood V. Vitamin C improves healing of foot ulcers: a randomised, double-blind, placebo-controlled trial. Br J Nutr. 2021;126(10):1451-1458. doi:10.1017/S0007114520003815

108. Kartika RW, Alwi I, Suyatna FD, et al. Wound Healing in Diabetic Foot Ulcer Patients Using Combined Use of Platelet Rich Fibrin and Hyaluronic Acid, Platelet Rich Fibrin and Placebo: An Open Label, Randomized Controlled Trial. Acta Med Indones. 2021;53(3):268-275.

109. Manning L, Ferreira IB, Gittings P, et al. Wound healing with "spray-on" autologous skin grafting (ReCell) compared with standard care in patients with large diabetes-related foot wounds: an open-label randomised controlled trial. Int Wound J. 2022;19(3):470-481. doi:10.1111/iwj.13646
